# Genome Evolution of Filamentous Cyanobacterium *Nostoc* Species: From Facultative Symbiosis to Free Living

**DOI:** 10.3390/microorganisms9102015

**Published:** 2021-09-23

**Authors:** Da Huo, Hua Li, Fangfang Cai, Xiaoyu Guo, Zhiyi Qiao, Weibo Wang, Gongliang Yu, Renhui Li

**Affiliations:** 1 CAS Key Laboratory of Algal Biology, Institute of Hydrobiology, Chinese Academy of Sciences, Wuhan 430072, China; huoda@ihb.ac.cn (D.H.); lih@ihb.ac.cn (H.L.); fangfangcai@whpu.edu.cn (F.C.); guoxiaoyu@ihb.ac.cn (X.G.); 2 Academy of Plateau Science and Sustainability, Qinghai Normal University, Xining 810016, China; 3 School of Animal Science and Nutritional Engineering, Wuhan Polytechnic University, Wuhan 430023, China; 4 University of Chinese Academy of Sciences, Beijing 100049, China; 5 Tianjin Key Laboratory of Aqua-Ecology and Aquaculture, College of Fisheries, Tianjin Agricultural University, Tianjin 300384, China; zhiyiqiao@tjau.edu.cn; 6 CAS Key Laboratory of Aquatic Botany and Watershed Ecology, Wuhan Botanical Garden, Chinese Academy of Sciences, Wuhan 430074, China; wangweibo@wbgcas.cn; 7 College of Life and Environmental Sciences, Wenzhou University, Wenzhou 325000, China

**Keywords:** cyanobacterial evolution, genome evolution, nostoc, adaptation

## Abstract

In contrast to obligate bacteria, facultative symbiotic bacteria are mainly characterized by genome enlargement. However, the underlying relationship of this feature with adaptations to various habitats remains unclear. In this study, we used the global genome data of Nostoc strains, including 10 novel genomes sequenced in this study and 26 genomes available from public databases, and analyzed their evolutionary history. The evolutionary boundary of the real clade of Nostoc species was identified and was found to be consistent with the results of polyphasic taxonomy. The initial ancestral species of Nostoc was demonstrated to be consistent with a facultative symbiotic population. Further analyses revealed that Nostoc strains tended to shift from facultative symbiosis to a free-living one, along with an increase in genome sizes during the dispersal of each exterior branch. Intracellular symbiosis was proved to be essentially related to Nostoc evolution, and the adaptation of its members to free-living environments was coupled with a large preference for gene acquisition involved in gene repair and recombination. These findings provided unique evidence of genomic mechanisms by which homologous microbes adapt to distinct life manners and external environments.

## 1. Introduction

Microbes play a major role in driving various ecological processes, and they contribute crucial functionalities to ecosystems [1,2]. They maintain multiple functions as autonomous biological entities or by forming symbionts that show high cooperation and low conflict with their partners [3,4,5]. Current studies have demonstrated that obligate symbiosis is strongly affected by genetic drift owing to the reduction of effective population size and biased genome deletion of redundant metabolic genes [6]. Furthermore, the additive effect of clonality and maternal transmission results in a high rate of fixation of slightly deleterious mutations [7]. Collectively, these factors generate a tendency of genome streamlining for an obligate symbiont [8,9]. However, microbial symbiosis can occur in various manners, except for obligate bacteria [10], some symbiotic microorganisms can associate with alternative hosts and even have a free-living stage during their life history (i.e., facultative symbiosis) [11]. Notably, recent studies have suggested that these microbes prefer a large genome size to meet metabolic requirements and thus adapt to a more complicated life cycle [12]. This inconsistent adaptation of microbial genomes—shifting from obligate symbiosis to free-living—warrants further investigation to gain insights into their underlying evolutionary mechanisms.

*Nostoc* species are a group of oxygenic phototrophic microorganisms composed of nodular filamentous cells within a gelatin-like sheath [13]. As well-known microbial pioneers in harsh terrestrial ecosystems, they share a range of particular characteristics when facing extreme conditions (e.g., drought and UV stresses), such as producing large amounts of exopolysaccharides [14] and synthesizing mycosporine-like amino acids [15]. Although *Nostoc* species, such as *N. sphaeroides* and *N. commune*, which have a strong resistance to stress, are usually free-living [16], other species can form a symbiotic relationship with their host plants [17,18]. The symbiosis between *Nostoc* species and terrestrial plants constructs a relatively stable microhabitat for the exchange of biochemical materials [19]. A particular trait of symbiotic *Nostoc* species is their facultative life cycle: they can undergo alternative life cycles involving both free-living cycles and forming an efficient symbiont with their hosts [20]. However, these genetically and morphologically similar *Nostoc* species that have symbiotic and free-living populations substantially differ in terms of genome size [21]. Thus, these *Nostoc* species provide an excellent research model for understanding the evolutionary associations of genomic enlargement with life history transformation.

The frequent errors committed in identifying *Nostoc*-like species via traditional taxonomy impede the advancement of genome research. Numerous filamentous cyanobacterial species that had been morphologically classified as *Nostoc* species were recently found to be dramatically different at the genetic level [22]. According to strict polyphased taxonomic analyses, a core portion of *Nostoc* s*ensu stricto* represents the real clade of *Nostoc* species (RNS hereafter) [23]. Moreover, to a large extent, the existing genomic data of *Nostoc* species lack rigorous definitions of their phylogenetic locations. Furthermore, most of the available genomic sequences of *Nostoc* species were obtained from symbiotic species [24]. In contrast to the 16S ribosomal RNA gene, comparative genomic studies of RNS, especially of free-living RNS, have been rarely conducted, resulting in confusion in the phylogenetic unification between free-living and symbiotic RNS and non-*Nostoc* species. Therefore, uncovering the underlying genomic mechanisms behind the ecological behaviors of *Nostoc* species is difficult.

In this study, we analyzed both free-living and symbiotic *Nostoc* strains to assess the influence of evolutionary transitions on the genome of this genus to adapt to diverse environments. According to the classification of *Nostoc sensu* *stricto*, we selected nine strains of free-living *Nostoc* and one strain of *Minunostoc*, which is a new genus of cyanobacteria recalibrated from *Nostoc*, for genome-wide sequencing. We also compared the sequenced genomes of *Nostoc* species recorded in the NCBI database. We performed molecular phylogenetic analysis and gene family analysis that focused on two evolutionary stages, namely, the formation of the real *Nostoc* and interspecies transitions from a symbiotic *Nostoc* to a free-living *Nostoc*. Results revealed a clear evolutionary boundary of real *Nostoc* species, which contains both free-living species and facultative endosymbionts. Genome amplification in the free-living population was found to be accompanied by the enrichment of functional genes related to gene repair and recombination during the dispersal of exterior branches.

## 2. Material and Methods

### 2.1. Sample Acquisition, Culture Condition, and Genome Sequencing

**Figure 1:**
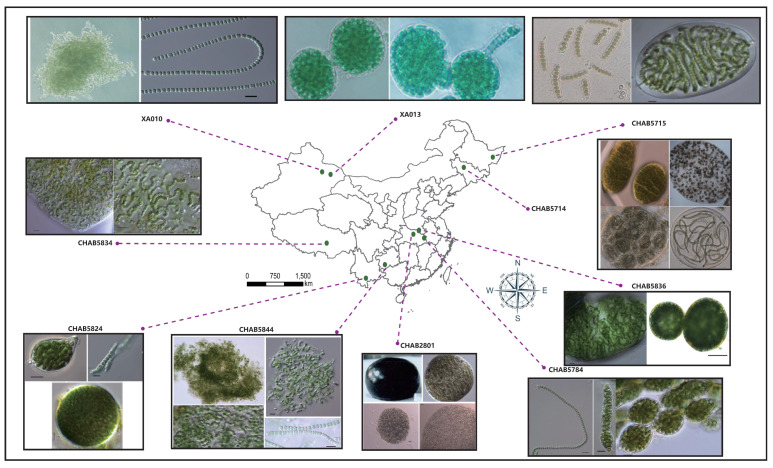
Geographical description of the isolation site. Violet dashed line linked the picture shows the morphological characteristics of each cultured strain.

*Nostoc*-like strains were collected from numerous sites in China from 2016 to 2017. The sampling sites are indicated in Figure 1. A total of 10 isolates, consisting of 9 RNS strains (i.e., *Nostoc* sp. CHAB5714, *Nostoc* sp. CHAB5715, *Nostoc* sp. CHAB5784, *Nostoc* sp. CHAB5824, *Nostoc* sp. CHAB5834, *Nostoc* sp. CHAB5836, *Nostoc sphaeroides* CHAB2801, *Nostoc* sp. XA010, and *Nostoc* sp. XA013) and 1 *Minunostoc* (a *Nostoc*-like species recently separated from *Nostoc*; *Minunostoc cylindricum* CHAB5844) [13] were sequenced. A single filament was separated under a microscope and cultured in a 25 °C constant-temperature incubator by using a solid and a liquid CT medium with a 12:12 light and dark cycle. Genomic DNA was extracted with PowerSoil DNA Isolation Kit (QIAGEN, GmbH, QIAGEN Strasse 1, Hilden, Germany). Whole-genome sequencing involved two different strategies: sequencing of the representative *Nostoc sphaeroides* CHAB2801 under the Pacbio sequel platform (Pacific Biosciences of California, Inc., Menlo Park, CA, USA) with a 10kb library and sequencing of the other strains under the Illumina Hiseq 2500 platform (Illumina, lnc., San Diego, CA, USA) with a paired-end 250 bp library.

### 2.2. Genome Assembly, Binning, Data Downloading, and Annotation

The FastQC v0.11.9 software was used for quality inspection of all sequencing reads [25], and low-quality reads were filtered with Trimmomatics [26]. Third-generation genomic sequencing was performed using the Canu v2.0 software with the default parameters chosen for assembly, with a specified genome length of 10 m and a correction depth of 200× [27]. A metagenome-binning strategy for the Illumina genome sequencing results was performed because of unavoidable bacterial contamination in extracellular polysaccharides of the *Nostoc* strains. We use Metaspades v3.12.0 software for metagenomic assembly [28]. The genome was assembled with mixed kmer lengths of 21, 33, 55, 77, 99, and 127, and the best assembly results were selected for subsequent analysis. The assembled contigs were regarded as a mixed pool that consisted of both the genome of *Nostoc* and bacterial contamination. The sequence of *Nostoc* in each binning result was extracted, using the COCACOLA binning pipeline to assign the assembled fragments into different genome bins. To improve the binning accuracy, we also used the Kraken v2.1.0 software to classify the binning results [29]. For each metagenomic sample, the results of the annotation tags containing Nostocales in other bins were transferred into the *Nostoc* bin. The second round of the assembly procedure was performed to obtain a high-quality binning result.

The original sequences were mapped into the merged binning results by using the BWA v0.7.17 software mem algorithm [30]. Finally, the spades v3.12.0 software [31] was used in the re-assembly to generate the final *Nostoc* genome scaffold results. The CheckM v1.1.3 software [32] was utilized to investigate the number and duplication of single-copy conserved genes in the whole genome and check genome integrity and redundancy. The genomic assembly results were separately annotated using the myRAST v33a22 software [33] and the Eggnog-mapper v2.0.5 software [34].

### 2.3. Gene Family Clustering, Phylogenetic Analysis, and Population Structure Evaluation

In the gene family clustering, the Diamond v2.0.6 software [35] was employed with the threshold E value of 10^−5^, and then the sequence in which the query and the identity results were <50% were excluded in the query result. The orthologous gene family was determined by using the OrthoMCL v2.0.9 software with a coefficient of expansion set to 1.5 [36]. The 16S rRNA gene sequences obtained in this study and those representing Nostocales cyanobacterial orders retrieved from GenBank were used in building a phylogenetic tree. Alignments were produced using MAFFT v7.312 [37], and a matrix of 125 sequences with 1048 nucleotide sites was finally formed. The phylogenetic tree was constructed using maximum-likelihood (ML) methods. The general time-reversible model (GTR) was selected as the optimal model for ML analysis, and ML algorithms were ran using PhyML 3.0 [38]. The core genome and the pan-genome of the algae and non-*Nostoc* algae were estimated, and then the average nucleotide similarity was determined using the *get_homologues.py* pipeline [39]. On the basis of the orthologous proteins, genome-wide phylogeny was inferred from 769 single-copy orthologous gene families in a concatenation tree. Using an in-house python script, the single-copy homologous protein family obtained from the clustering results from the entire protein sequence was extracted. Subsequently, a single copy of the homologous amino acids of each strain was aligned with muscle software. Bad alignments were then removed with Gblocks v0.91b [40]. Finally, all the alignments were connected into a super protein sequence. The whole-genome phylogeny was constructed using RaxML v8.2.12 [41] with the PROTGAMMAWAGF model. The population structure of *Nostoc sensu stricto* was further investigated by, performing a Bayesian analysis of population structure (i.e., BAPS) [42] to identify genetically different groups. Genome alignment was performed with ProgressiveMauve v2.4.1 [43], and then the core alignment was extracted with the script stripSubsetLCBs v2.4.1 and then concatenated using a custom Python script. Principal components analysis (PCA) was conducted using Plink [44], and downstream files for BAPS were generated. After filtering bad singleton single nucleotide polymorphisms (SNPs), the hierarchical manner software (hierBAPS v1.0.0) was used to assign the *Nostoc* strains into a distinct genetic lineage with two hierarchical scales.

### 2.4. Inferring Ancestral Genome Content and Statistic

The evolutionary history of *Nostoc* was further investigated by reconstructing ancestral gene family sizes by using the COUNT software [45] with a birth-and-death model. The model was described by lineage- and family-specific gene loss and duplication rates and coupled with a family gain process to account for the arrival by horizontal gene transfer. All analytical procedures were performed using R v3.5.1 [46].

## 3. Results

### 3.1. Genomic Assembly and Binning Results

Owing to the lack of accurate classification data in the NCBI database, we had to calibrate the RNS species before we could investigate the genomic features of *Nostoc.* Over the past few years, we had collected and cultured morphologically and ecologically identified *Nostoc* species from different habitats in China. Molecular analysis revealed that only a 12 strains were actually RNS genetically. In this study, we sequenced nine isolates of RNS and one *Minunostoc* species, and both were morphologically and genetically identified strictly. The geographic information of the sampling sites is described in Figure 1. A detailed description of the morphological characteristics of RNS is provided in Supplementary Material Table S1. The results of genomic assembly and binning are given in Supplementary Material Table S2. The genomes of other *Nostoc*-like species were obtained from the NCBI database labelled “*Nostoc*.” A detailed explanation is provided in Supplementary Material Table S3. In all assembly results, each genome size was >7 M. The biggest genome was that of CHAB5784, with a full length of 9.8 M. The number of gene-encoding proteins in the genome was relatively large compared with that of other cyanobacteria as all of which were more than 8000 genes. The maximum number of genes was 10,676, and the GC content of the assembly results was approximately 42%. The genome of *N*. *sphaeroides* CHAB2801 was assembled into a complete genome by using the Pacbio RSII platform.

### 3.2. Phylogenetic Reconstruction

**Figure 2:**
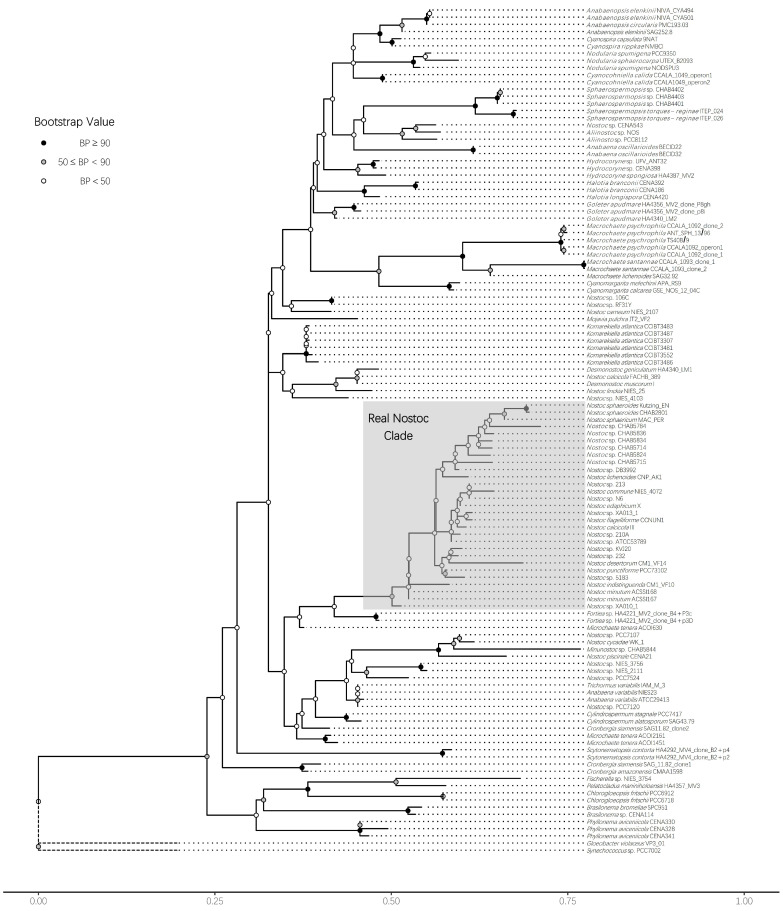
Coordinates of RNS in Nostocales. A maximum-likelihood phylogenetic tree of *Nostoc*-like strains based on the 16S rRNA gene was constructed, and the topological structure was tested with 1000 replicated bootstrap tests in Raxml. The RNS is marked in the gray shade, and the bootstrap value is shown on each node of the tree.

**Figure 3:**
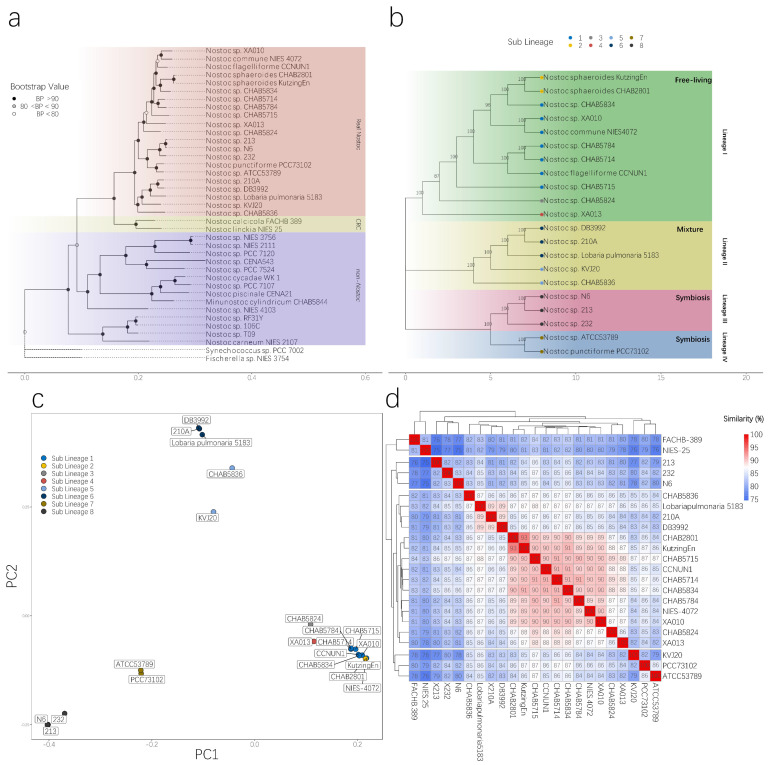
Phylogenetic differentiation of RNS and non-*Nostoc* species. (**a**). Maximum likelihood phylogenetic tree reconstruction of the core genome of 37 strains of the genome of *Nostoc*-like strains by using the Raxml software. A GTRGAMMA model was adopted in the analysis, and 100 bootstrap tests were performed. The number on each node represents the bootstrap value. (**b**). *Nostoc* was divided into four clades and marked in different colors by Bayesian analysis of population structure. (**c**). Principal component analysis of the shared SNPs among RNS genera, (**d**). Average nucleotide identity (ANI) among *Nostoc* and CRC strains. The color gradient shows the ANI value of each genome pair. The final analysis results were plotted using the ggplot2 v3.1.0 software package [47].

To obtain a reliable phylogenetic topology and satisfy the requirements of different analyses, we adopted different approaches to building the phylogenetic tree. Finding a reliable labeled strain is easier with 16S rRNA gene information. Thus, to locate the position of real *Nostoc* species in the phylogenetic tree, we built a 16S rRNA gene tree via the ML method. We defined RNS according to the modern taxonomic theory. The species should have 97% similarity in 16S rRNA gene with the type species (*Nostoc commune* in *Nostoc* genus). All species in the genus should be single-originated in phylogenetic structure. The results confirmed the identification of RNS and non-*Nostoc* strains we sequenced in this study. Several strains labeled as *Nostoc* by other studies were out of the range of the RNS clade, indicating that numerous records of *Nostoc* in the NCBI database were the result of incorrect identification (Figure 2). We then built the phylogenetic tree by using the ML method to concatenate whole-genome homologous single-copy protein sequences. Results showed that the phylogenetic trees could clearly distinguish RNS from non-*Nostoc* algae, thereby supporting the findings of the phylogenetic tree constructed using 16S rRNA gene. Notably, two isolates of the FACHB-389 and NIES-25 strains were closely related to the RNS groups. We named this branch the Closely Related Clade (CRC). On the basis of the 16S rRNA gene tree, we deducted that these two strains most likely belong to the *Desmonostoc* branch. The other isolates, including *Minunostoc* CHAB5844, were distinctly out of the RNS range (Figure 3a).

### 3.3. Population Structure of RNS Species

We further described the genetic relationship within the RNS clade via a hierarchical Bayes test. In the third phylogenetic tree we built for BAPS analysis, we used whole-genome SNPs conducted by Raxml. Our estimation of the population indicated that the RNS species analyzed herein contain four lineages and at least eight sub-lineages. Except for CHAB5836, the individuals we sequenced all belong to clade I. CHAB5836 belongs to lineage II (Figure 3b). The topology structure we generated via different methods supported a robust phylogenetic relationship between RNS and non*-Nostoc* species. Each sub-lineage represents a different lifestyle. All strains from lineage I were isolated from free-living *Nostoc* species. Lineages III and IV indicated a symbiotic lifestyle. Lineage II contains both the free-living strain CHAB5836 and the other symbiotic strains. PCA using whole-genome SNP information supported the results of BAPS and depicted the relationship between the strains more intuitively. The free-living species from the clusters in lineage I were tightly herded, indicating that they were highly similar. We also performed an average nucleotide identity (ANI) analysis (Figure 3d). With regard to the pair-wise nucleotide similarity, only the isolates in lineage I had an average similarity of 89.13% ± 1.45%. Two CRC clade strains could be clearly distinguished from RNS in the ANI analysis, indicating that CRC underwent a certain degree of genetic differentiation. Unexpectedly, *Nostoc sphaeroides* CHAB2801 and Kutzing En (16S rRNA gene similarity 100%) showed the highest similarity in the ANI analysis, reaching 93.48%, but also exceeded the traditional definition of prokaryotic species, which states that interspecies ANI distance should be within 95%–96% [48]. KVJ20 in lineage II had a higher ANI with lineage IV species than in other lineage species. Moreover, the species in lineage III formed a distinct cluster with other species.

### 3.4. Distinct Genomic Traits of RNS and Free-Living Nostoc Species

**Figure 4:**
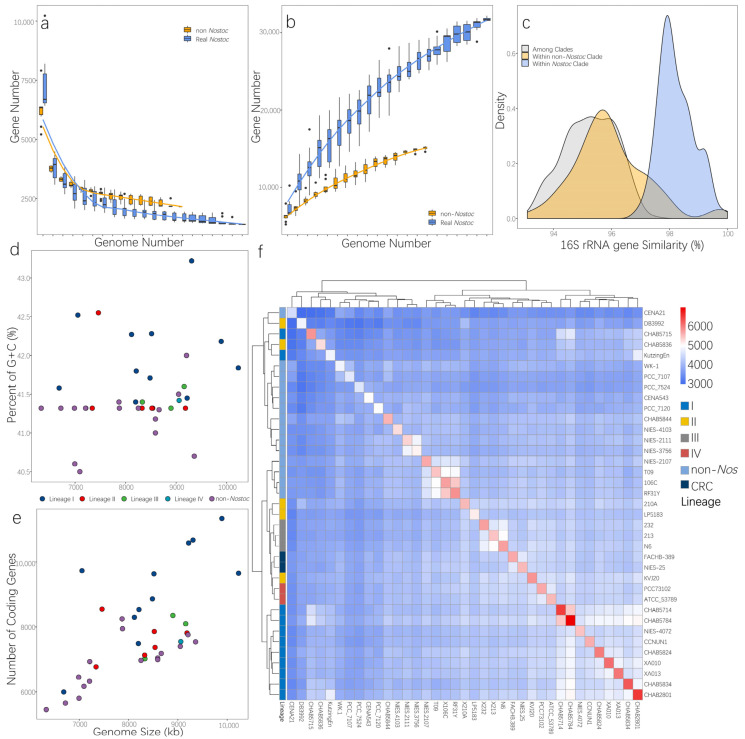
Comparison of genomic characteristics of RNS and non-*Nostoc* species. The core pan-genomes of RNS and non-*Nostoc* species were estimated. (**a**). Core genome. (**b**)**.** Pan-genome. (**c**)**.** Density plot of paired-wise comparison of 16S rRNA gene similarity. Comparison of (**d**) G + C ratio on each strain (**e**) genome size versus the number of coding genes. (**f**). Shared number of ortholog proteins of each strain. The strains with high similarity were clustered together via the hclust method. Each color lump on the rows represents the phylogenetic clade of each strain, and the clustering results were counted and plotted using the Pheatmap package in R [49].

The genome content of RNS and non-*Nostoc* species were compared. Results showed that no specific gene was shared among the RNS species analyzed herein (Supplementary Material Table S4). Nevertheless, apparent differences were observed in terms of the ability to integrate foreign genes in the RNS strains. We estimated the core genome and the pan-genome of the RNS and non-*Nostoc* strains. As shown in Figure 4a,b, the core genome curve of non-*Nostoc* strains converged to 2245 genes after the addition of the 13th genome. The core genome of RNS was less than that of non-*Nostoc* species and converged to 1414 genes when the 21st genome was added. (The estimated genome size was 1787.6 ± 176.074 when added to 13 genomes.) A smaller core genome was observed in RNS. Interestingly, compared with the pan-genome curves, the pan-genome size of the algae rapidly increased with the addition of new genomes, and the number of non-*Nostoc* species was considerably fewer than that of *Nostoc* species. Genomic variation analysis revealed differentiation between RNS and non-*Nostoc* species, in which RNS harbored both a higher GC content and a larger genome (Figure 4d,e). The free-living strains from lineage I had a higher GC content and genome size. Unexpectedly, *Nostoc* Kutzing En, which was assigned to clade I, was only 6.6 MB large. The paired-wise matrix of shared ortholog proteins illustrated that most strains of RNS clustered together (Figure 4f). However, four strains, namely, DB3992, CHAB5715, CHAB5836, and Kutzing En, were inserted in the non-*Nostoc* clade. Meanwhile, two strains from CRC had more ortholog proteins with the RNS clade. In general, the topology from the shared protein matrix was inconsistent with the phylogenetic tree generated herein. Except for CHAB5715 and Kutzing En, all the other strains from the free-living clade in lineage I formed a distinct cluster with the other strains.

### 3.5. Reconstruction of Ancestral Genome

**Figure 5:**
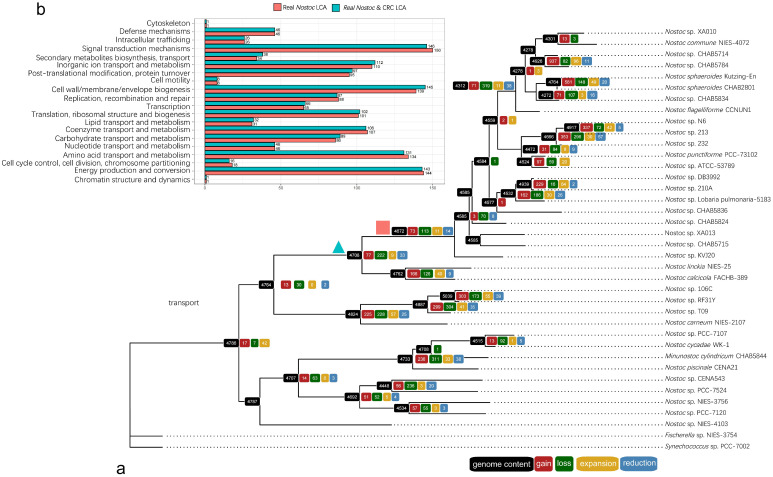
Inferring ancestral genome content by using the Count software. The inference relies on the topology of a single-copy ribosomal protein tree made by Raxml. (**a**)**.** Gene family numbers involved in evolutionary changes are shown on the tip of each ancestor position. (**b**). Comparison of the genome of the last common ancestor (LCA) of CRC and RNS. The green triangle represents the LCA of both CRC and RNS, and the pink square represents the LCA of RNS.

**Figure 6:**
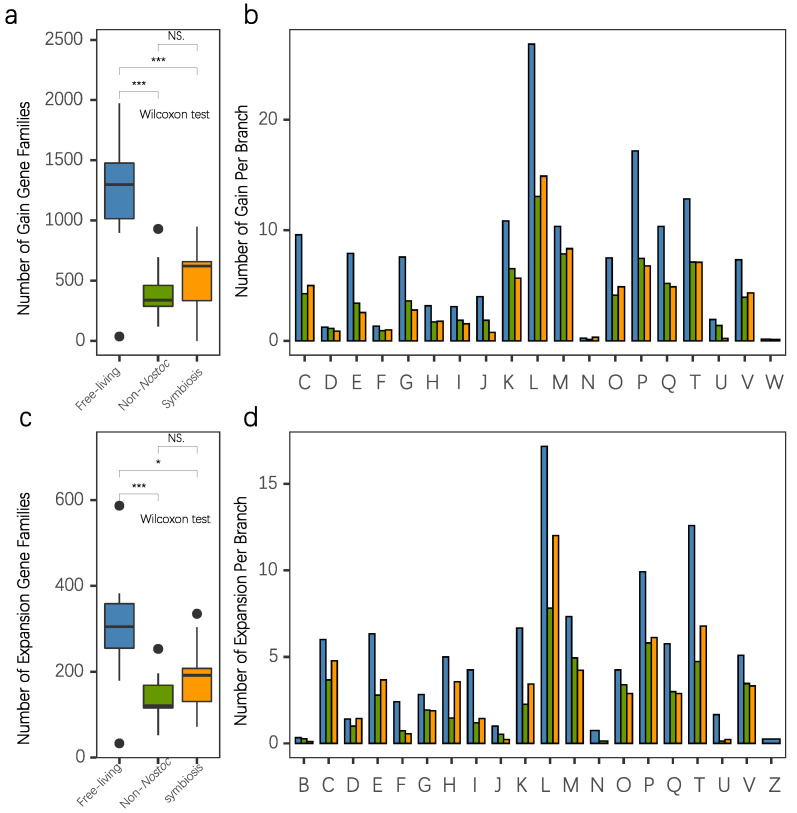
Evolutionary events related to genome enlargement in the exterior branch. Average gene family of (**a**) gain and (**b**) expansion (increasing paralog). (**c**,**d**). Gene family gained per branch among free-living *Nostoc* (FL), symbiotic *Nostoc* (SN), and non-*Nostoc* species (NN). Asterisks indicate significant differences based on Wilcoxon test (* *p <* 0.05 *** *p <* 0.001).

The genome content of the ancestor of every evolutionary stage was also inferred via the ML method under the birth-and-death model [50]. We identified 4672 gene families predicted to be present in the last common ancestor (LCA) of RNS and 4708 gene families for the LCA of RNS and CRC. By comparing the two ancestor genomes, that is, the LCA of RNS and CRC, we found only small differences in the clusters of orthologous groups (COG), which is related to the reduction of cell wall/member/envelope biogenesis capacity and the amplification of signal transduction (Figure 5b). We investigated the genes present in the ancestral genome of RNS (pink square in Figure 5a) but absent in the ancestral genome of CRC and RNS (blue triangle in Figure 5a). The results are provided in Supplementary Material Table S5. These genes were more likely required for the formation of RNS, except for 12 proteins of unknown function (S). The acquired families, along with the formation of RNS toward four families of amino acid transport and metabolism, four families of replication recombination and repair, three families of cell wall/membrane/envelope biogenesis, and three families of energy production and conversion. The genes absent in the genome of the LCA of RNS but present in the ancestor were probably lost during the formation of RNS (Supplementary Material Figure S1). A high proportion of lost families was involved in six families of cell wall/membrane/envelope biogenesis; four families of replication, recombination, and repair; and a small number of other families with diverse functions. By comparing different evolutionary events, we found that events associated with genomic amplification were more likely to have occurred in the exterior branch. The events involved in genome amplification (acquisition and expansion) occurred more frequently in the exterior of the free-living species (Supplementary Material Figure S2). The COG annotation of these events is given in Figure 6. In the free-living branch, gene acquisition and expansion were biased toward the function of replication, recombination, and repair; inorganic ion transport and metabolism; and signal transduction. The number on each tip indicated the number of gene flows that happened. The evolutionary event related to genome amplification (gain and expansion) mainly happened to the common ancestor of the sub-lineage in RNS, which occurred more often than genome reduction events (loss and contraction).

## 4. Discussion

Previous researchers argued that the genomic evolution of *Nostoc* might have relied on several divergent events [51]. However, modern cyanobacterial taxonomy holds the viewpoint that all the verified species should come from a single common ancestor or lineage, even for prokaryotic species. Such an understanding of the taxonomy of any cyanobacteria species is difficult to achieve because of misidentification [52] and the lack of robust evidence supporting their taxonomy. Some studies on the genome of *Nostoc* and related genera emphasized that their taxonomy requires a comprehensive revision [53]. How many strains in the NCBI database are actually *Nostoc* strains remains an open question. This study was the first to unify the taxonomy and genomic evolutionary history of *Nostoc*. Our results supported the strict definition of the taxonomic unit of *Nostoc*. By using 16S rRNA gene sequences from numerous finely identified strains, we were able to determine the phylogenetic position of RNS (Figure 2). Unsurprisingly, numerous strains in the NCBI database with the registered name of *Nostoc* have a distant genetic distance to the real *Nostoc* species. Further analysis with genome trees supported this observation. A strain that was previously considered to have numerous similar signatures in morphological appearance can easily differ from RNS in both the genome and 16S rRNA gene trees. We further investigated the population structure of the RNS by introducing a Bayesian algorithm to the analysis with a hierarchical annotation of phylogenetic clades. The results indicated that RNS has a diverse community structure. The structure had at least four main lineages and eight sub-lineages in the current RNS group (Figure 3b). PCA analysis based on the core genome SNP information supported the clustering results from BAPS analysis. These subtle branches corresponded to the evolution of RNS from a symbiotic relationship to a free-living lifestyle. Therefore, RNS includes a vast diversity of species.

We also observed phylogenetic incongruence in the results. The strains from lineages I and II frequently exchanged positions in different phylogenetic trees probably because of genomic recombination that happened during the evolutionary history of *Nostoc*. To minimize the impact of incongruent phylogeny, we generated a fourth phylogenetic tree by concatenating 23 ribosomal proteins. The dividing line of RNS and non-*Nostoc* species consisted of every phylogenetic tree, thereby supporting our definition of RNS as a relatively reproductively isolated unit.

According to the biological species concept (BSC), individuals of the same species should be able to mate and breed specifically with each other [54]. However, the strict BSC is difficult to use in defining a prokaryotic species because of occasional HGT and homologous recombination among different microbial groups [55]. Contemporary taxonomic ideas state that each taxonomic unit should harbor the species that owns a common ancestor (monophyletic) [56]. However, reaching an actual identification of cyanobacteria is a challenging task because of its vast diversity, minor signature of appearance, and frequent convergent evolutionary events [57]. In contrast to cyanobacteria, ecological responses, biochemical reactions, and molecular markers are widely used in studying the taxonomy of other bacteria because of the complete loss of dissimilarity in appearance. Taxonomic research based on morphological divergence has set the standards for defining cyanobacterial species for a long time, and it has had a profound effect on research on cyanobacteria. Filamentous algae with a similar appearance but with a distinct genetic discrepancy comprise a high proportion of cyanobacterial species. If previous studies only focused on the full range of ecological effects or environmental communities of cyanobacteria, then the results could be accepted. However, when these results are applied in investigating evolutionary variations or genetic mechanisms, they will result in confusion unless the taxonomy of cyanobacteria is accurately described. Therefore, research on the genome of cyanobacteria should focus on the phylogenetic and ecological relationship of each strain. The present study provided sufficient evidence obtained from primary genomic features to gene flow patterns that RNS and previously identified *Nostoc* species are remarkably different. Such an excess number of foreign genes was gained in the common ancestor of each sub-lineage of RNS, and the presence of only a rare ortholog gene shared with non-*Nostoc* species indicated that most gene acquisition was transferred from external species and suggested a gene flow barrier between non-*Nostoc* species and RNS. Recent studies attempted to reconcile the contradiction in identifying cyanobacteria on the basis of morphology by using genome information. The well-known bloom-forming cyanobacteria *Microcystis*, which cannot be identified by a specific single gene marker, has a higher inter-clade recombination rate than that between clades [55]. Resolving problems in phylogenetic relationships may be difficult because of genome hybridization. Nevertheless, this scenario can only happen within a specific genetic distance range. If the phylogenetic relationship is too far, even among species that have a similar appearance and share several homologous genes, then those species should not be considered to be the same species.

Various studies highlighted the impact of symbiosis on the evolution of prokaryotic species [58,59,60]. *Nostoc* is a famous symbiont with different lifestyles: they can be free-living, an extracellular symbiont, or a facultative symbiont. After revising the definition of RNS, we found that the clade of RNS corresponded to facultative symbionts, which can form intracellular symbiosis. Obligate symbionts always experience the progress of genome streamlining because of genetic drift and low effective population size (*N*_e_), which are a result of reducing the capacity of metabolism by natural selection. Recent work on a marine cyanobacterium, *Prochlorococcus*, suggested that the procedure seems to be a neutral process as this bacterium contains numerous non-conservative amino acid changes during its evolution [61]. Previous studies on *Nostoc azollae* indicated that this strain underwent genomic streamlining [62]. The present study suggested that *N. azollae* does not belong to RNS because the common ancestor of RNS is capable of intracellular symbiosis. Thus, RNS has a larger genome than non-*Nostoc* species. Genomic signature analysis did not reveal any specific gene that belongs to the present RNS group (Supplementary Material Table S4) nor a small core genome with a large pan-genome (Figure 4a,b). More gene gains that happened in the sub-lineage clades (Figure 5) indicated that the expansion of genome size occurred during the dispersal of these sub-lineages and that genes were acquired from different donors. This result was consistent with that of previous studies, which reported that facultative symbiotic algae seem to have undergone genomic amplification and have accumulated more gene family gains than losses, suggesting that cyanobacterial genomic amplification may be associated with facultative symbiosis [53]. We also found that the nonsymbiotic RNS had a higher GC content (Figure 4c). A high GC content is observed in species that can grow in freezing and desiccating environments [63], consistent with the ecological fitness of RNS. Aside from the changes in the basic features of the genome, we also found that the gene dynamics events that occurred at the ancestor nodes of *Nostoc* were closely related to their adaptive symbiotic lifestyle. The gene acquisition events related to the formation of RNS were mainly associated with five biological processes, namely, biosynthesis of lipopolysaccharides, formation of algal hormogonia, photosynthesis, damage repair, and resistance enhancement, which are characteristics of RNS. Three genes, namely, *Wzm*, *rfbF*, and *rfbC*, were found to have been gained in the LCA of RNS, which corresponded to the biosynthesis of lipopolysaccharides. Many Gram-negative bacteria use lipopolysaccharide molecules as the position indicator of the cell membrane porins for ion and molecule exchange. These three genes reportedly play essential roles in the synthetic procedure of lipopolysaccharides [64,65,66]. Some studies also uncovered the functions of these genes in the symbiotic relationship between bacteria and their host plants. In *Herbaspirillum seropedicae*, *rfbB* and *rfbC* disrupt the ability of this bacterium to attach onto the surface of maize root; the attachment was 100-fold lower than that in wild-type maize, suggesting that lipopolysaccharides are required by *H*. *seropedicae* to attach to maize root and to colonize plant tissues internally [66]. Nostocaceae cyanobacteria always produce few gliding filaments, which are termed hormogonia, as part of their life cycle [67]. As an efficient movement method, hormogonia serve as a dispersal agent and a plant infection agent. The LCA of RNS was found to have gained two genes, namely, *cheB* and *pilL*, which are related to hormogonium formation. The products of *cheB* are essential in producing changes in swimming direction and flagellar rotation in *Escherichia coli*. The homolog genes of *cheB* in cyanobacteria are related to hormogonium formation [68], and it reportedly has multiple copies in the genome of the symbiotic *N. punctiform* [21]. Pilus-like appendages are expressed on the surface of hormogonia. Mutations in pil-like genes alter piliation and reduce symbiotic competency [69]. Two genes related to photosystem I, namely, *psaB* and *psaJ*, were also found to have been gained in the genome of the LCA of RNS. Five genes related to damage repair and resistance enhancement, namely, *AlkB*, *gstA*, *Pre*, *lon*, and *yfdZ*, as well as other genes related to biological functions, such as *nifV*, facilitate bacterial symbiosis and nitrogen fixation. The acquisition of these genes supported the results of the phylogenetic analyses of the intracellular symbiotic status of the ancestor of RNS.

**Figure 7:**
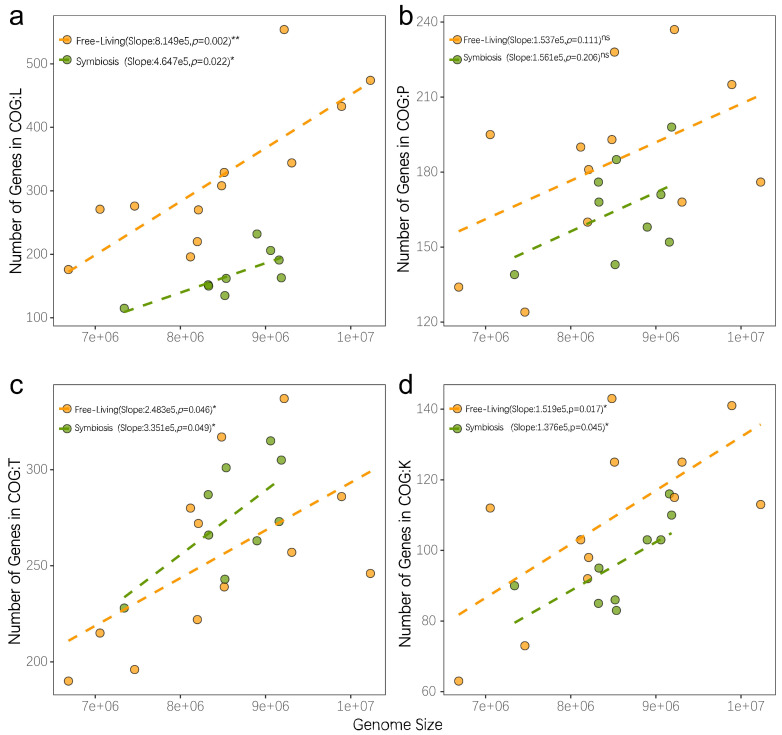
*Nostoc* genome enriches different genome functions under different lifestyles. Yellow point indicates the genome with free-living lifestyle and green point for symbiotic lifestyle. The dashed line shows the linear regression relationship between genome size and the number of coding genes in each COG type. Each figure indicates genes functions belonging to (**a**) COG L Replication and repair, (**b**) COG P Inorganic ion transport and metabolism, (**c**) COG T Signal Transduction, and (**d**) COG K Transcription.

Evolution is a continuous process of change to adapt to a wide range of living environments. Aside from the changes induced by mutations in essential evolutionary forces, the exchange of genetic material between species is also an important method for altering microbial genomes. Many studies showed that species acquire genetic traits that improve fitness through horizontal gene transfer to adapt to a particular environment. In this study, we observed changes in genome size and codon preference among *Nostoc* species with similar genetic distances. The RNS community exhibited an extensive pan-genomic profile that demonstrated the robust genomic plasticity of RNS. Further analysis of genome reconstruction revealed that these exogenous genes are involved in many different functions. Traditional studies on the adaptation of microbial genomes to the environment often focused on changes in microbial metabolic functional diversity. Our study found that the evolution of free-living *Nostoc* was accompanied by a dramatic increase in genome size (Figure 6). In general, the genomic enlargement caused by microbial adaptation to a wide range of environments is due to the integration of exogenous genes, leading to the improvement in microbial metabolic functional diversity. However, we found that, although horizontal gene transfer plays a substantial role in the adaptation of *Nostoc* to a wide range of free-living environments that results in a remarkable increase in genomic size, the increase in the number of genes is diverse and not limited to metabolic functions. The coding density of most functional genes is similar in the symbiotic *Nostoc* community as it is in the free-living *Nostoc* species. The slope was only notably shifted by the increase in functional genes involved in genome recombination and repair (Figure 7). Free-living *Nostoc* species often face harsh conditions, such as ultraviolet exposure and extreme water scarcity. The damage to DNA caused by these environmental conditions is a common challenge to terrestrial cyanobacteria. A previous study found a notable increase in the number of genes involved in gene repair in the genome of *N. flagelliforme* [14]. Our study further revealed that extensive acquisition of gene repair functions is a common feature of free-living *Nostoc* species.

In conclusion, by considering 16S rRNA gene similarity with type species, phylogenomic analysis supports a separate evolutionary clade of RNS, which is consistent with previous single-gene studies. In addition, even if they are similar in morphological observations, RNS shows higher genomic plasticity by sharing a larger pangenome but similar 16S rRNA gene among species. Population structure analysis revealed that RNS contains different groups of free-living and symbiotic species. The free-living RNS exhibited high genomic plasticity to adapt to different environments. Evolutionary patterns of *Nostoc* provide an infrequent case that experienced a dramatic increase in genome size within similar phylogenetic groups. We propose that intracellular symbiosis is an essential procedure for the formation of RNS genera, and free-living *Nostoc* species have evolved to acquire more genes involved in gene repair and recombination to adapt to diverse environments.

## Data Availability

Genomic sequences of the new sequenced *Nostoc* genomes are available at the NCBI GenBank database under the accession number PRJNA668662.

## Supplementary Materials

The following are available online at https://www.mdpi.com/article/10.3390/microorganisms9102015/s1, Table S1: Morphological description of the sequencing strains, Table S2: Genome assembly and binning results, Table S3: Basic information of downloaded sequences in this research, Table S4: The genes present in the genome of the NSS closest common ancestor but missing in the previous ancestor, Table S5: The genes present in the genome of the common ancestor of NSS and CRC but missing in the NSS ancestor, Figure S1. Pangenome analysis of all available genomes, Figure S2: Evolutionary events happened along the phylogeny of Nostoc and other Nostoc-like species.
