# Supplementary material for: Genome Evolution of Filamentous Cyanobacterium Nostoc Species: From Facultative Symbiosis to Free Living
Source: Microorganisms. 2021 Sep 23;9(10):2015. doi: 10.3390/microorganisms9102015 (PMC8539589; doi:10.3390/microorganisms9102015)
Supplement: Supplementary file 1 [file microorganisms-09-02015-s001.zip › microorganisms-1357503-supplementary.pdf]

microorganisms

Table S1. Morphological description of the sequencing strains.

Sequencing Strains	Morphological Description
**Nostoc sphaeroides CHAB2801**	Colonies are spherical, soft, colloid, with smooth surface and solid periphery. Solitary colonies are usually up to 1-4 cm in diameter. Filaments flexuous, irregularly aggregated. Vegetative cells are short barrel-shaped or nearly spherical, about 4.55 μm long and about 4.7 μm wide, terminal cells often slightly elongated and narrowed up to 2.5 μm. Heterocytes are nearly spherical, 6.2-7.3 μm in length and 5.4-6.6 μm in width. The akinetes cell wall is smooth and spherical.
**Minunostoc cylindricum** CHAB5844	Colonies macroscopic, gelatinous, irregularly clustered, free–living on wet rocky wall in the form of irregular, amorphous, blue–green gelatinous clusters. Filaments flexuous, freely entangled. Sheath usually present, colourless. Vegetative cells barrel–shaped, 3.1– (4.3)–5.4 μm long, 2.5– (3.2)–3.8 μm wide, length: width ratio 1.3. Heterocytes and akinetes were not observed.
***Nostoc* sp. XA013**	Colonies microscopic. In the early stage of life, the filaments were very densely aggregated, could not be seen clearly. In the later stage, the filaments arranged loosely, and the filament broke up into short filaments, and then the spheres burst to release shorter filaments. The end cells of filaments are longer than wide, 2.26-3.34 μm in length, short-barrel to long-barrel vegetative cells, 1.46-2.15-2.80 μm in length and 2.07-2.42-2.80 μm in width. Heterocytes are short barrel-shaped, larger than vegetative cells, about 3.97 μm long and 4.47 μm wide. No akinete was found.
***Nostoc* sp. XA010**	Colonies are gelatinous, irregularly clustered. Filaments are usually enveloped by gelatinous sheath. Vegetative cells are short barrel-shaped, 3.15 μm long and 3.51 μm wide. Heterocytes are larger than vegetative cells, long barrel-shaped, 4.81 μm long and 4.34 μm wide. No akinete was found.
***Nostoc* sp. CHAB5714**	Several small spherical colonies are enveloped by a large colony, and the large colony surface are smooth with firm periderm. Filaments are densely entangled especially in young colonies. Vegetative cells are short barrel-shaped to nearly spherical, or long oval, about 3.09 μm in length and 4.43 μm in width. Heterocytes are nearly spherical, similar in size to vegetative cells, with a diameter of 4.3 μm. No akinete was observed.
Nostoc sp. CHAB5715	Colonies are spherical, microscopic. In the early stage of life, small colonies are aggregated into a long filamentous colony. Old colony are solitary. Trichome are enveloped by a thick, colorless sheath. Vegetative cells are short barrel-shaped, 4.19 μm long and 5.29 μm wide. Heterocytes are nearly spherical, slightly larger than vegetative cells, with a diameter of 5.99 μm. No akinete was observed.
***Nostoc* sp. CHAB5784**	Colonies are spherical, microscopic. Young colonies are connected with each other by the heterocyte, filaments are densely entangled in colonies. Single trichome are enveloped by sheath or not. Vegetative cells are oval or spherical, about 3.36 μm in length and 3.50 μm in width, and the ratio of length to width is 0.96. Heterocytes are slightly larger than vegetative cells, usually short barrel-shaped, about 3.89 μm in length and 4.06 μm in width. No akinete was observed.
***Nostoc* sp. CHAB5836**	In young colonies, filaments are closely wound, filaments adhere to the firm sheath, and then to form a large spherical colony. The vegetative cells of a single filament are cylindrical, and the vegetative cells in the colony are short barrel-shaped, 3.48 μm long and 3.68 μm wide. Heterocytes are larger than vegetative cells, nearly spherical or short barrel-shaped, 4.10 μm long and 4.50 μm wide, with a length-width ratio of 0.91. No akinete was observed.
***Nostoc* sp. CHAB5824**	Colonies are spherical, microscopic. heterocyte are at both end of the young, oval colony. Filaments are densely entangled in colonies.Vegetative cells are oval, 1.82-4.11-5.14 μm long and 1.82-2.56-3.58 μm wide. Heterocytes and vegetative cells are similar in size, short barrel-shaped, 2.44-3.04-3.95 μm long and 2.33-2.99-3.73 μm wide. No akinete was observed.
***Nostoc* sp. CHAB5834**	Colonies are spherical, macroscopic, with loose and irregular filaments. Vegetative cells are spherical to short barrel-shaped, 1.77-2.35-2.95 μm long and 2.03-2.63-3.43 μm wide; heterocytic,

spherical or long elliptic, slightly larger than vegetative cells, about 3.58 μm in length and 3.66 μm in width, no akinete was observed.

Table S2. Genome assembly and binning results.

Organism name	Length	Contigs	GC (%)	Proteins	Completeness	Contamination
N. favosum CHAB5714	8,514,377	245	42.28	9637	100	1.89
N.exigua CHAB5715	7,054,926	1201	42.52	9732	87.62	3.41
N.mirabile CHAB5784	9,892,412	297	42.18	11341	99.92	4.19
N.tenue CHAB5824	8,117,724	138	42.27	8296	99.7	2.42
N.rigida CHAB5834	9,307,052	563	43.22	10676	100	7.82
N.pachydermaticum CHAB5836	7,461,718	384	42.55	8549	99.11	2.78
N.minutum XA010	8,210,661	137	41.80	8536	100	1.56
N.xinjiangensis XA013	8,483,714	211	41.71	8865	100	6.9
N.sphaeroides CHAB2801	9,217,956	11	41.45	10585	Complete	Complete
Minunostoc cylindricum CHAB5844	7,868,568	208	41.40	8248	99.04	1.41

Table S3. Basic information of downloaded sequences in this research.

Organism name	GenBank number	Length	contigs	GC (%)	Proteins	Completeness	Contamination
*N.*sp. 213	1168001	8,331,440	380	41.4	7021	99	0.74
*N.*sp. 232	1167991	9,158,766	398	41.6	8097	98.78	1.37
N.commune NIES-4072	1693891	8,198,700	44	41.40	7489	99.77	0.22
*N.*sp. ATCC 53789	1822731	8,536,834	220	41.32	7370	99.56	0.44
N.calcicola FACHB-389	916171	9,049,214	354	41.5	7401	99.22	1.11
N.piscinale CENA21	488611	7,094,556	11	40.5	6172	96.99	0.44
*N.*sp. PCC 7120	31208	7,211,789	7	41.32	6931	99.19	0
*N.*sp. PCC 7107	502788	6,329,823	1	41.32	5456	99.26	0.33
*N.*sp. PCC 7524	502768	6,718,869	3	41.32	5655	99.33	0
*N.*sp. CENA543	1532881	7,207,640	6	41.32	6329	99.78	0.22
*N.*sp. 5183	1592541	7,339,908	5	41.32	6769	99.55	0.44
N.linckia NIES-25	1232421	8,590,040	21	41	7042	99.89	0.89
*N.*sp. NIES-2111	1232511	7,876,755	11	41.32	7945	99.3	0.11
*N.*sp. KVJ20	786451	9,184,886	425	41.32	7813	99.67	0.89
*N.*sp. 210A	1168011	8,325,913	73	41.32	7130	99.22	1.33
*N.*sp. NIES-3756	635661	6,987,571	3	41.32	6451	99.3	0.11
*N.*sp. DB3992	1368581	8,521,473	1,037	41.32	7860	94.54	4.6
*N.*sp. RF31Y	1110281	9,205,737	629	42	7759	99.11	2.61
N.cycadae WK-1	1533001	6,994,461	150	40.6	5803	99.56	0.89
*N.*sp. NIES-4103	1232571	8,590,607	50	41.18	6994	99.26	0.44
N.carneum NIES-2107	1232481	9,359,223	16	40.70	7541	99.33	0.19
*N.*sp. 106C	1110051	8,662,725	241	41.3	7184	99.11	1.44
*N.*sp. T09	1110041	8,251,853	314	41.32	6969	99.11	0.33
N.punctiforme PCC73102	41068	9,059,191	6	41.42	7548	99.56	0.44
N.sphaeroides Kutzing En	1912231	6,684,490	5	41.58	5998	99.48	0
*N.*sp. N6	1592491	8,898,590	11	41.32	8353	99.44	1.19
N.flagelliforme CCNUN1	1460651	10,231,233	9	41.84	9655	100	3.07

Table S4. The genes present in the genome of the NSS closest common ancestor but missing in the previous ancestor.

Gene Name	COG	Function
alkB	L	Alkylated DNA repair protein
ALL0363	L	Transposase
RC1_0998	L	Transposase
AVA_2181	L	Inherit from COG: transposase
pre	S	plasmid recombination enzyme
wzm	V	Transporter
PLAV_1218	S	methylase helicase
BSL1696	S	WGR domain-containing protein
nifV	E	Homocitrate synthase
psaB	C	PsaA and PsaB bind P700, the primary electron donor of photosystem I (PSI), as well as the electron acceptors A0, A1 and FX. PSI is a plastocyanin cytochrome c6-ferredoxin oxidoreductase, converting photonic excitation into a charge separation, which transfers an electron from the donor P700 chlorophyll pair to the spectroscopically characterized acceptors A0, A1, FX, FA and FB in turn. Oxidized P700 is reduced on the lumenal side of the thylakoid membrane by plastocyanin or cytochrome c6
PilL	T	CheA signal transduction histidine kinase
PsaJ	C	May help in the organization of the PsaE and PsaF subunits (By similarity)
YfdZ	E	Aminotransferase
OCAR_5339	S	oxidoreductase
folE	H	GTP cyclohydrolase I
mtnD	S	Catalyzes 2 different reactions between oxygene and the acireductone 1,2-dihydroxy-3-keto-5-methylthiopentene (DHK-MTPene) depending upon the metal bound in the active site. Fe-containing acireductone dioxygenase (Fe-ARD) produces formate and 2-keto-4- methylthiobutyrate (KMTB), the alpha-ketoacid precursor of methionine in the methionine recycle pathway. Ni-containing acireductone dioxygenase (Ni-ARD) produces methylthiopropionate, carbon monoxide and formate, and does not lie on the methionine recycle pathway (By similarity)
cheB	T	catalyzes the demethylation of specific methylglutamate residues introduced into the chemoreceptors (methyl-accepting chemotaxis proteins) by CheR (By similarity)
PDEN_1306	D	Cobyrinic acid ac-diamide synthase
ceaA	P	efflux system protein
AZL_008610	S	Phage protein Gp37/Gp68
ssuD	S	Monooxygenase
ndh	C	FAD-dependent pyridine nucleotide-disulfide oxidoreductase
gstA	O	Glutathione S-transferase N-terminal domain protein
metX	E	homoserine O-acetyltransferase
mutT	F	Nudix hydrolase
SCLAV_5486	S	Inherit from bactNOG: Cupin 2, conserved barrel domain protein
BL01679	E	SAM-dependent methyltransferase
SCLAV_2440	S	ABC transporter
ycgL	S	Predicted nucleotidyltransferase
lon	O	Inherit from bactNOG: ATP-dependent Lon protease
yaiS	S	GlcNAc-PI de-N-acetylase
rfbF	M	Glucose-1-phosphate cytidylyltransferase
umaA	M	cyclopropane-fatty-acyl-phospholipid synthase
SSCG_04708	S	protein containing aminopeptidase domain
rfbC	M	dTDP-4-dehydrorhamnose 3,5-epimerase

Supplementary material S6

Table S5. The genes present in the genome of the common ancestor of NSS and CRC but missing in the NSS ancestor.

Gene Name	COG	Function
GLL1611	L	Transposase
dnaB	L	Replicative DNA helicase
AVA_1381	L	Transposase IS66
NblA	S	phycobilisome degradation protein
CheB2	T	CheB methylesterase
AppA	E	Extracellular solute-binding protein, family 5
AqpZ	M	Channel that permits osmotically driven movement of water in both directions. It is involved in the osmoregulation and in the maintenance of cell turgor during volume expansion in rapidly growing cells. It mediates rapid entry or exit of water in response to abrupt changes in osmolarity (By similarity)
BMUL_4716	S	ATP-binding protein
gumM	M	Glycosyl transferase WecB TagA CpsF
yvkB	K	Transcriptional regulator, TetR family
spoT	K, T	HDc
igiC	Q	Asp Glu hydantoin racemase
lpcA	M	Glycosyl transferase family 8
wcaF	M	Inherit from bactNOG: colanic acid biosynthesis acetyltransferase wcaF
SCLAV_1551	S	Methyltransferase type 11
HTH_0578	S	Phosphatidylethanolamine-binding protein
mtnB	G	Catalyzes the dehydration of methylthioribulose-1- phosphate (MTRu-1-P) into 2,3-diketo-5-methylthiopentyl-1- phosphate (DK-MTP-1-P) (By similarity)
dmpI	S	4-oxalocrotonate tautomerase
BL05203	E	Amino acid permease
PolB	L	DNA polymerase
OCAR_6107	M	Glycosyl transferases group 1
rsgA	S	May play a role in 30S ribosomal subunit biogenesis. Unusual circulary permuted GTPase that catalyzes rapid hydrolysis of GTP with a slow catalytic turnover (By similarity)
rbpD	S	RNA-binding protein
purM	F	phosphoribosylaminoimidazole synthetase
bchE	C	hopanoid biosynthesis associated radical SAM protein HpnJ
ypfJ	S	Putative neutral zinc metallopeptidase
tauB	P	ABC transporter
PPS	G	pyruvate phosphate dikinase PEP pyruvate-binding
yccU	S	CoA_binding
rplL	J	Seems to be the binding site for several of the factors involved in protein synthesis and appears to be essential for accurate translation (By similarity)
sbP	S	56kDa selenium binding protein (SBP56)
OCAR_5127	S	Sulfite exporter TauE/SafE
pyrE	F	Catalyzes the transfer of a ribosyl phosphate group from 5-phosphoribose 1-diphosphate to orotate, leading to the formation of orotidine monophosphate (OMP) (By similarity)
modA	P	ABC transporter, periplasmic molybdate-binding protein
MC7420_3283	T	RecA-superfamily ATPases implicated in signal transduction
SCLAV_3941	O	PHB
cpsF	M	Oligosaccharide biosynthesis protein Alg14 like
pssE	S	Glycosyltransferase 28 domain protein

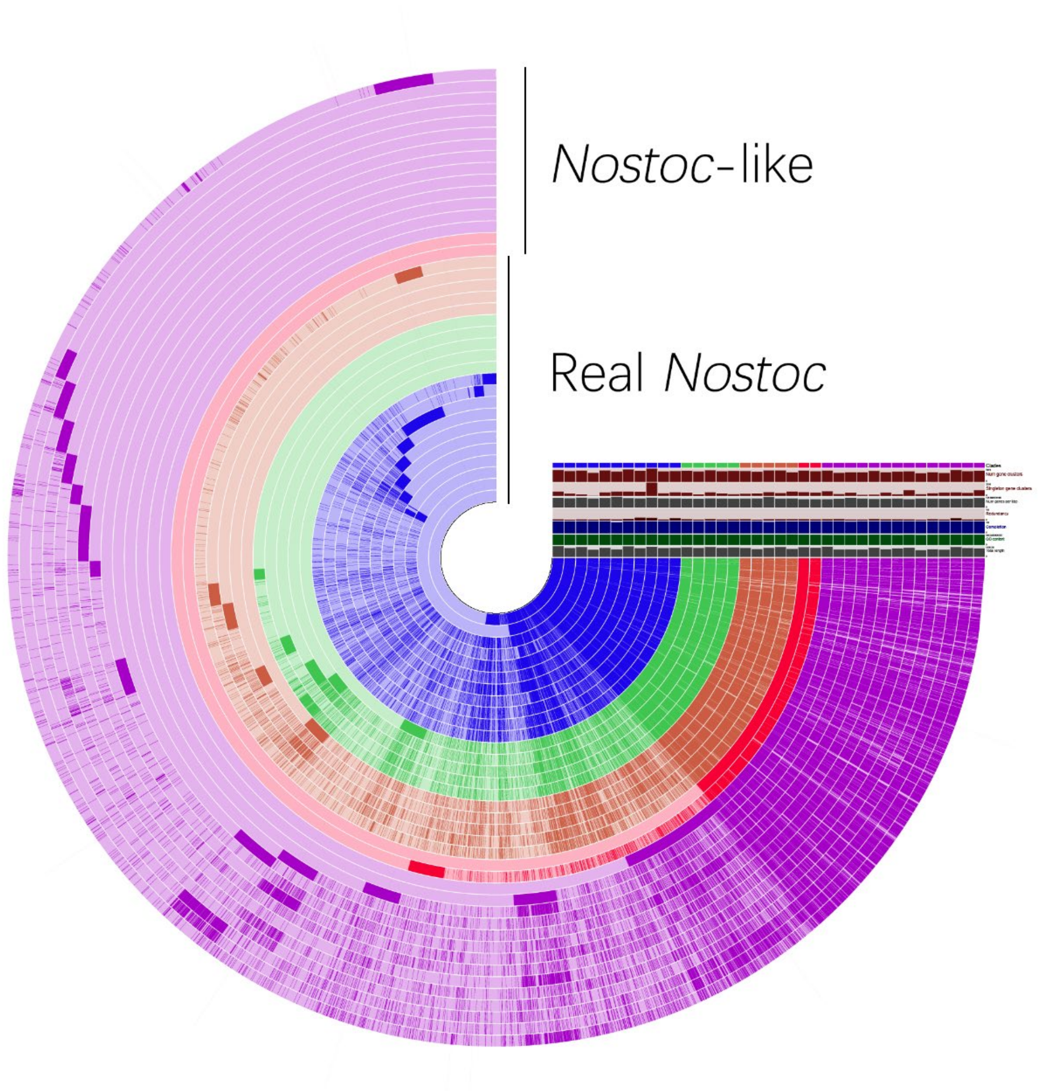

Figure S2. Pangenome analysis of all available genomes.

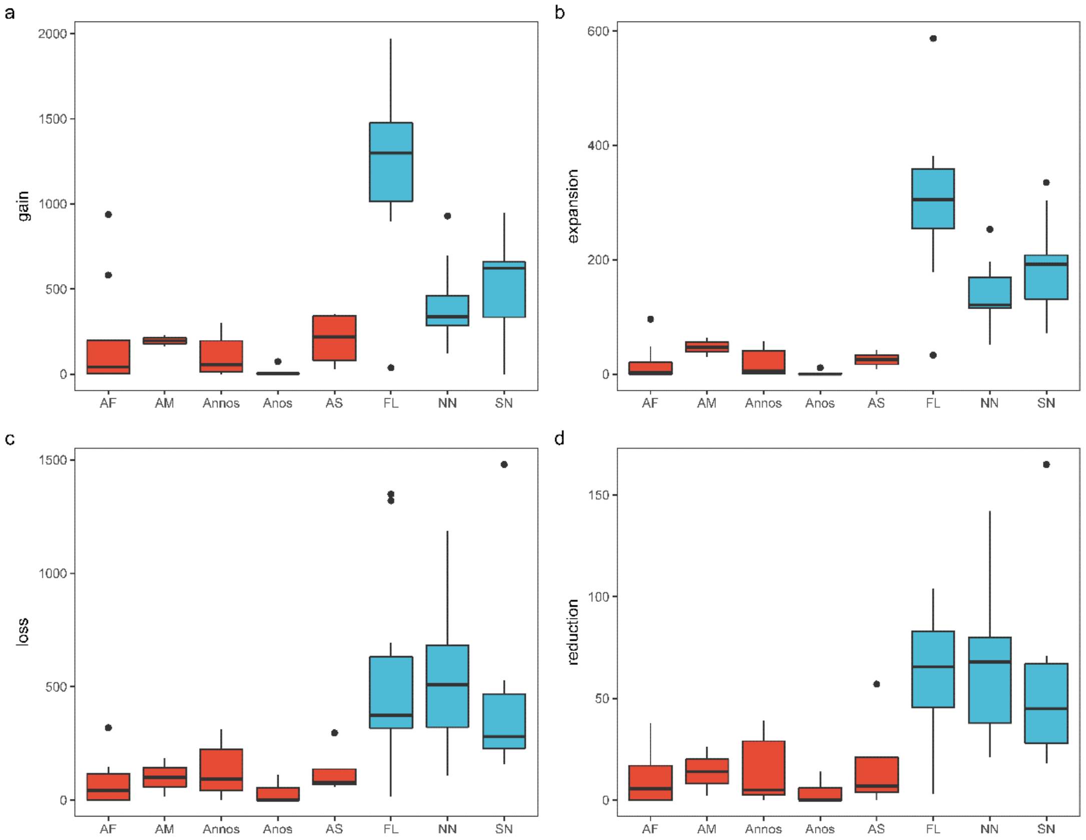

Figure S2. Evolutionary events happened along the phylogeny of Nostoc and other Nostoc-like species.AF:Ancestor of Free-living *Nostoc* **AM:**Ancestor of mixture clades Annos: Ancestor of Non-Nostoc species(ie., *Nostoc*-like species) **AS:**Ancestor of Real Nostoc species **FL:**Free-living nostoc **NN:**Non-Symbiosis Nostoc **SN:** Symbiosis nostoc.
